# Fusion level selection in posterior hemivertebra resection for congenital scoliosis: clinical effect size driven feature selection and multi-criteria optimization decision model

**DOI:** 10.3389/fmed.2026.1919903

**Published:** 2026-09-11

**Authors:** Mincheng Zou, Yuhao Yang, Ya Liu, Feng Yao, Xiaodong Wang, Yong Qiu, Fuyong Zhang

**Affiliations:** 1 Department of Orthopaedics, Children's Hospital of Soochow University, Suzhou, Jiangsu, China; 2 Department of Orthopaedics, Nanjing Drum Tower Hospital, Nanjing, Jiangsu, China

**Keywords:** congenital scoliosis, decision tree model, hemivertebra, segmental fusion, spinopelvic alignment, surgical strategy

## Abstract

**Study design:**

Retrospective study.

**Objectives:**

There is a lack of objective criteria for the selection of fixed segment length in hemivertebra resection and fusion. This study aimed to develop and validate decision models to guide the selection of the best surgical strategy, based on radiographic parameters.

**Methods:**

Thirty six patients who underwent posterior hemivertebra resection and fusion were included. Preoperative, postoperative and follow-up imaging data were integrated, and the key predictors were screened by effect size. Multi-weight threshold analysis was used to determine the optimal decision threshold of each factor. Classification and regression tree (CART) algorithm was used to construct the prediction model of fusion strategy selection, and the performance was evaluated by leave-one-out cross validation.

**Results:**

Lumbar lordosis, thoracolumbar kyphosis, and pelvic tilt were identified as predictors to distinguish segments fusion. Model was stratified according to locations of the hemivertebra, with an accuracy of 93.5% and an area under the curve (AUC) of 0.982. The core predictors maintained stable discriminative power across anatomical subgroups.

**Conclusions:**

The decision tree model based on preoperative imaging can effectively distinguish the patients with hemivertebra deformity requiring long- and short-segment fusion, which provides an objective decision-making tool for optimizing the surgical strategy, balancing the correction effect and function preservation.

## 1 Introduction

Hemivertebra is one of the most common causes of congenital scoliosis (1). It is caused by a unilateral vertebral developmental defect caused by an embryonic vertebral formation disorder (2).This anatomical anomaly usually leads to wedge-shaped deformity, which in turn causes progressive scoliosis and kyphosis. Without effective intervention, the deformity may continue to worsen, not only affecting the appearance, but also leading to serious complications such as trunk imbalance, impaired cardiopulmonary function and nerve compression.

Currently, posterior hemivertebra resection with fusion and internal fixation has been widely considered as the gold standard for the treatment of this disease (3), which can effectively remove teratogenic lesions and rebuild spinal stability. However, the length of fusion segment is still controversial in clinical practice, and the core is how to achieve a balance between orthopedic effect and functional preservation. In current clinical work, short-segment (two-segment) fusion (SSF) has become the mainstream choice because it can maximize the preservation of spinal motor function and growth potential (4, 5), especially for young patients (6, 7). However, when facing patients with larger or more rigid scoliosis, longer fusion segments (more than two segments) may provide better kyphosis correction and long-term stability (8). At the same time, the location of the hemivertebra (thoracic, thoracolumbar, or lumbar) also affects the surgical approach and the choice of fusion segment (1).

The dilemma in decision-making is that insufficient fusion may lead to loss of correction or aggravation of compensatory curve, while excessive fusion may unnecessarily sacrifice spinal motor function and affect the long-term quality of life of the patient. At present, the selection of fusion level largely depends on the personal experience and preference of the surgeon, and there is a lack of objective and quantitative decision support tools.

Therefore, by integrating preoperative radiographic parameters and postoperative correction effect, using effect size based feature selection method, combined with multi-criteria decision analysis, our study developed and validated a data-based decision model, aiming to objectively quantify the surgical indications and identify patients suitable for long-segment fusion (LSF) or SSF, so as to provide a reliable decision aid tool for clinical individualized treatment.

## 2 Methods

### 2.1 Study design and patient selection

Patients with single hemivertebra deformity of congenital scoliosis who underwent surgical treatment in our center from March 2019 to March 2024 were retrospectively selected. Surgical indications were determined according to previous literature (9). All operations were performed by the same spinal deformity specialist. All surgeries were performed via a posterior midline approach with the patient prone. Neuromonitoring was performed throughout the operation. Complete hemivertebra resection was performed, followed by posterior spinal fusion and pedicle screw fixation. Complete preoperative, postoperative and follow-up imaging data were obtained.

The following exclusion criteria were set: (1) multiple hemivertebra and/or combined with other spinal deformities; (2) absence of imaging data at any time point or follow-up time less than 24 months. (3) concomitant systemic diseases (e.g., neuromuscular scoliosis, connective tissue disease, skeletal dysplasia) that may independently affect the surgical strategy or postoperative spinal mechanics evolution. (4) severe postoperative complications (infection, internal fixation-related complications, neurological complications, and secondary surgical intervention due to failed correction). Complications may interfere with the postoperative rehabilitation trajectory, so that the observation of orthopedic effects cannot simply reflect the biological and mechanical effects of the surgical fixation strategy itself.

Finally, 36 patients were enrolled. The study protocol was approved by the ethics committee (No. 2025CS320), and written informed consent was obtained from guardians of all participants.

### 2.2 Data collection and variable definition

The baseline data, radiographic parameters and surgical information of the patients were systematically collected. Baseline data included age, sex, height, and weight. Hemivertebra features include location and type. Surgical information included operation time, blood loss, and length of hospital stay. Radiographic evaluation mainly focused on coronal plane balance, major curve Cobb, head side compensatory Cobb, rear side compensatory Cobb, coronal sacral inclination angle, segmental kyphosis, sagittal plane balance, pelvic tilt angle (PT), thoracic kyphosis angle, thoracolumbar kyphosis angle (TLK), lumbar lordosis angle (LL), sagittal sacral inclination angle and other indicators. All parameters were measured at three standardized time points: preoperative, early postoperative (1 month after surgery), and the last follow-up (≥24 months after surgery) to comprehensively evaluate the dynamic changes of spinal balance.

The main outcome measures included the type of fusion segment [long segment (>2 segments) and short segment (two segments)], radiological improvement rate, and maintenance rate of correction effect.

### 2.3 Statistical analysis

Continuous variables are presented as interquartile ranges. Shapiro-Wilk test was used to assess normality, Wilcoxon rank-sum test was used, and Cliff's delta effect size (δ) was calculated. Cliff's delta = [#(long segment value > short segment value) – #(long segment value < short segment value)]/(N_length × N_short), evaluating |δ| < 0.147 as a negligible effect and 0.147 ≤ |δ| < 0.33 as a small effect. 0.33 ≤ |δ| < 0.474 was a moderate effect, and |δ| ≥ 0.474 was a large effect. Odds ratios were used to assess group differences for categorical variables, and Cramer's V coefficient was used to measure the strength of association between variables, with the criteria of 0.1 ≤ V < 0.3 as small effect, 0.3 ≤ V < 0.5 as moderate effect, and V ≥ 0.5 as large effect.

### 2.4 Intervention effect evaluation and feature selection

The evaluation indicators of intervention effect included improvement rate and maintenance rate. The correction rate = (preoperative value -postoperative 1 month value)/preoperative value × 100%, reflecting the early postoperative correction effect. The maintenance rate = (last follow-up value – 1 month value after operation)/(preoperative value – 1 month value after operation) × 100%, reflecting the long-term stability of the correction effect. Both were evaluated for statistical significance by Wilcoxon test (*p* < 0.05).

Our qualitative maintenance rate >100% was defined as “further improvement”; 90%−100% were “excellent maintenance”; 80%−89% were “well-maintained”; 70%−79% were “general maintenance”; < 70% was considered as “poor maintenance”; A negative value indicates “worse than before” Wilcoxon test was used to compare the improvement rate and maintenance rate between the two groups, and Cliff's delta effect size was calculated.

To comprehensively evaluate the predictive value of feature variables, a feature comprehensive scoring system was constructed: Composite score = Cliff's delta (preoperative) × 0.6 + Cliff's delta (improvement rate) × 0.25 + Cliff's delta (maintenance rate) × 0.15, and the effect size of the improvement rate and maintenance rate of the inclusion criteria were both ≥-0.2. On this basis, the feature priority was evaluated, including high priority (composite score > 0.4), medium priority (0.3 ≤ composite score ≤ 0.4), and low priority (0.2 ≤ composite score < 0.3). Finally, 2–3 features with high priority or the highest comprehensive score were selected as the model input.

### 2.5 Weight sensitivity analysis and threshold selection

A weight sensitivity analysis was performed on the selected characteristic factors to assess the impact of different weight assignments on the final threshold selection and to determine the range of weights capable of producing stable thresholds. The combination of test accuracy weight (0.2–0.7, step 0.1), coverage weight (0.2–0.7, step 0.1) and case number weight (0, 0.05, 0.1) was analyzed, and the sum of weight recombination was 1. Overall score = accuracy × Weight_acc + coverage × Weight_cov + (proportion of cases) × Weight_samp, and the optimal threshold was the value that maximized the overall score. The “stable weight range” of each feature was identified based on the threshold change of no more than ±10% of the median.

The comprehensive evaluation was based on three different weighting strategies. The balanced model (accuracy 0.5 + coverage 0.45 + sample size 0.05) was designed to balance the accuracy and sensitivity of the model. Conservative type (accuracy 0.65 + coverage 0.3 + sample size 0.05) prioritized prediction accuracy to avoid overtreatment; the sensitive type (accuracy 0.4 + coverage 0.55+ sample size 0.05) focused on identifying LSF cases to reduce the risk of missed diagnosis. All the strategies required that the number of cases below any threshold be at least 3 or 30% of the total positive cases to ensure statistical stability.

Precision = true positive/(true positive + false positive), Recall/Sensitivity = true positive/(true positive + false negative). Clinical recommendation criteria were defined. Accuracy ≥60% and coverage ≥70% were considered as strong recommendation, accuracy ≥50% and coverage ≥60% were considered as recommendation, accuracy ≥40% and coverage ≥50% were considered as caution or not recommended alone.

### 2.6 Model construction and evaluation

CART algorithm was used to construct the decision tree prediction model. Aiming at the problem of small samples and class imbalance (long/short = 7:29), the loss matrix was set, and the cost of misclassification of long segment to short segment was set as three times of the misclassification cost of short segment to improve the recognition ability of minority classes.

The position of hemivertebra was used as a covariable to conduct stratified modeling, compare different segmentation characteristics, importance ranking of variables and differences in decision rules, evaluate the necessity of classification, and ensure the clinical applicability of the model.

The performance of the model was evaluated by multi-dimensional indicators such as accuracy, recall (coverage), precision and specificity. The stability and generalization ability of the model were evaluated by leave-one-out cross-validation (LOOCV). McNemar test was used to compare the consistency of the prediction results of different models. The change rate of decision was analyzed from the perspective of clinical transformation, and the impact of the new model on actual treatment decisions was evaluated. The biological rationality of the model decision was explored by comparing the distribution of characteristics. Receiver operating characteristic (ROC) curve analysis was used to evaluate the discriminant efficiency and clinical diagnostic value of the model.

Finally, the out-of-sample prediction probability obtained by LOOCV was used to calibrate the model. To quantify the sampling variability of model performance, we employed the Bootstrap resampling method (1,000 iterations with replacement) to calculate the 95% confidence intervals (CIs) for all performance metrics. The percentile method was used to estimate the lower and upper bounds of the CIs, thereby reflecting the robustness of the point estimates under the limited sample size.

To evaluate the clinical utility of the model, we performed decision curve analysis (DCA). DCA calculates the net benefit across a range of threshold probabilities and compares the model's predictive strategy with the strategies of treating all or no patients. The model was considered clinically useful if its net benefit exceeded that of both extreme strategies across clinically reasonable threshold ranges.

### 2.7 Software and tools

All statistical analyses and prediction models were constructed using R (version 4.4.3, R Foundation for Statistical Computing, Vienna, Austria). The main software programs included rpart (decision tree modeling), pROC [ROC curve and area under the curve (AUC) calculation], caret (cross validation and model training), and ggplot2 (Wickham, H. Springer-Verlag New York) (visualization). *p* < 0.05 was considered statistically significant. Data with missing values were excluded, and a fixed random seed was set during the analysis to ensure the reliability and reproducibility of the results. TRIPOD+AI guidelines was used for systematic evaluation of the models.

## 3 Results

### 3.1 The difference of radiographic parameters before operation and convergence after operation

A total of 36 patients were included, including seven patients in the LSF group and 29 patients in the SSF group. The baseline data of each group were shown in Table 1. The radiographic parameters of each group were shown in Supplementary Table S1. Compared with SSF group, the LSF group had significantly greater scores in several core sagittal parameters (SPs), including TLK (19° vs. 9.5°, *p* = 0.013), LL (66° vs. 45.5°, *p* = 0.02), segmental kyphosis (22° vs. 13°, *p* = 0.045) and thoracic kyphosis (40° vs. 21°, *p* = 0.045). The effect size was moderate or above (Cliff's delta > 0.33). In addition, patients in the LSF group were older (12.75 vs. 4.83 years, *p* = 0.004), and the procedure time was significantly longer (205 vs. 165 min, *p* = 0.02). There were no significant differences in gender, hemivertebral type and other radiographic parameters between the two groups (Tables 1, 2).

**Table 1:** Comparison of baseline characteristics between patients with long- and short-segment fusion.

Variable	Category	Long_segment (percentage/median_IQR)	Short_segment (percentage/median_IQR)	*p*_value	Effect_size	Effect_label
Gender	Male	5 (71.4%)	18 (62.1%)	1	0.65	OR
	Female	2 (28.6%)	11 (37.9%)			
Location	T1–T10	3 (42.9%)	4 (13.8%)	0.087	0.369	Cramér's V
	T11–L2	4 (57.1%)	15 (51.7%)			
	L3–L5	0 (0%)	10 (34.5%)			
Type	FS	5 (71.4%)	19 (65.5%)	0.868	0.089	Cramér's V
	SS	2 (28.6%)	9 (31%)			
	NS	0 (0%)	1 (3.4%)			
Age		12.75 (11, 13.42)	4.83 (3.75, 8.08)	0.004	0.719	Cliff's_delta
Height		148 (145.25, 156)	110 (97, 121)	0.019	0.638	Cliff's_delta
Weight		47 (34.7, 48.1)	19 (15.5, 24.9)	0.024	0.562	Cliff's_delta
Operation_time		205 (192.5, 242.5)	165 (135, 194)	0.02	0.576	Cliff's_delta
Blood_loss		400 (350, 600)	275 (200, 412.5)	0.134	0.372	Cliff's_delta
Hospital_stay		14 (13, 15)	15 (13, 19)	0.856	−0.049	Cliff's_delta

NS, non-segmented; SS, semi-segmented; FS, fully-segmented.

**Table 2:** Comparison of radiographic parameters between patients with long- and short-segment fusion.

Variable	Long_before (median_IQR)	Short_before (median_IQR)	*p*_value_before	Cliff's_delta_before	Long_last (median_IQR)	Short_last (median_IQR)	*p*_value_last	Cliff's_delta_last
Coronal_plane_balance	7.35 (5.74, 10.85)	9.59 (4.48, 13.09)	0.869	−0.046	1.15 (0, 12.91)	9.96 (4.98, 16.79)	0.199	−0.324
Major_curve_Cobb	36 (32.5, 36.5)	32 (27, 36)	0.575	0.143	13 (11, 18.5)	11 (6.5, 14)	0.24	0.296
Head_side_compensatory_Cobb	12 (7.5, 14.5)	12 (6, 16)	0.841	−0.054	12 (8.5, 16)	6.5 (4.25, 9.75)	0.107	0.407
Rear_side_compensatory_Cobb	24 (19.5, 25)	13 (8.5, 17.25)	0.009	0.653	4 (2, 12.5)	3 (1, 7)	0.582	0.143
Coronal_sacral_inclination_angle	5 (0.5, 5.5)	5.5 (1.75, 11.25)	0.136	−0.372	3 (1, 5)	3 (1, 7.5)	0.577	−0.143
Segmental_kyphosis	22 (14, 27)	13 (9, 18)	0.045	0.503	18 (15.5, 22.5)	16 (8.5, 19)	0.193	0.328
Sagittal_plane_balance	30.18 (16.23, 39.67)	27.3 (11.44, 40.02)	0.8	0.071	28.2 (10.3, 41.19)	18.31 (11.83, 29.4)	0.682	0.11
Pelvic_tilt_angle	9 (4.5, 10)	13.5 (8, 22.25)	0.065	−0.47	6 (3, 16)	8.5 (2.75, 14.25)	1	0.006
Thoracic_kyphosis_angle	40 (26, 48)	21 (13.25, 28.75)	0.045	0.505	36 (27.5, 41.5)	30 (17, 34)	0.09	0.429
Thoracolumbar_kyphosis_angle	19 (14.5, 36)	9.5 (5.5, 18)	0.013	0.621	13 (8, 16)	8 (4.5, 12.5)	0.294	0.265
Lumbar_lordosis_angle	66 (55.5, 74.5)	45.5 (37, 58.25)	0.02	0.588	60 (53.5, 62)	47 (34.5, 53)	0.047	0.497
Sagittal_sacral_inclination_angle	39 (34.5, 45)	35.5 (29.25, 41)	0.311	0.258	33 (31, 38.5)	36 (28.5, 40)	0.932	0.026

**Figure 1:**
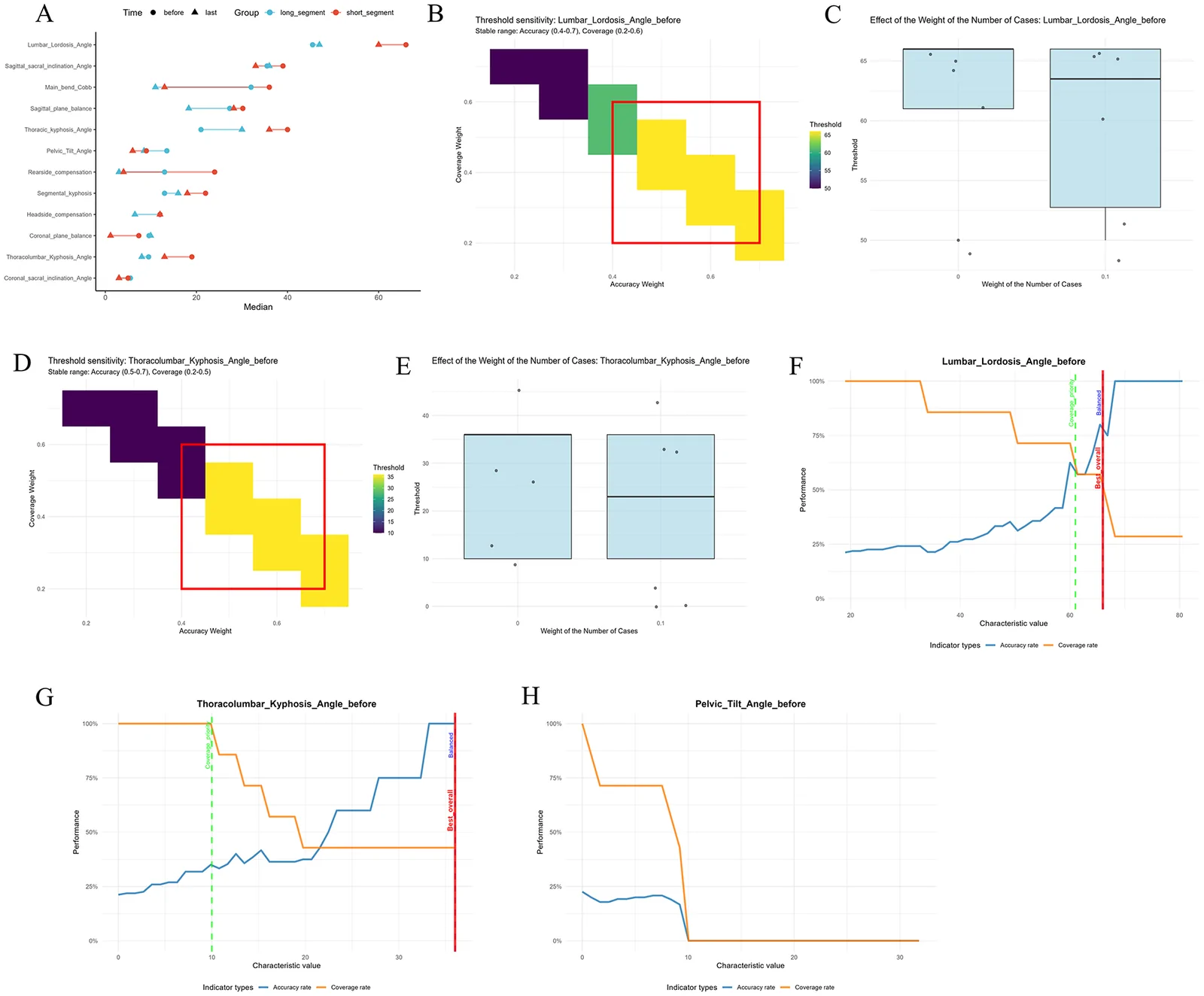
Weight optimization of key features and comprehensive performance evaluation. **(A)** The changes of the main radiographic parameters of the two groups before surgery and at the last follow-up. **(B, C)** Stable range of weights for accuracy rate, coverage rate and sample size of the lumbar lordosis angle feature. **(D, E)** Stable range of weights for accuracy rate, coverage rate and sample size of the thoracolumbar kyphosis angle feature. **(F, G, H)** Accuracy rate, coverage rate and the best comprehensive weight selection of lumbar lordosis angle, thoracolumbar kyphosis angle and pelvic tilt angle as single-factor key features under different thresholds (the sample size weight is all 0.05).

At the last follow-up, the radiographic parameters showed significant improvement in both groups, and the differences between the groups in the above parameters disappeared (*p* > 0.05), with the between-group effect size decreasing to a negligible level (Cliff's delta < 0.147; Figure 1A).

### 3.2 Coronal improvement advantage of SSF and sagittal maintenance trend of LSF

A comparison of postoperative improvement and maintenance rates revealed the unique efficacy characteristics of the two surgical strategies (Tables 3, 4).

**Table 3:** Differential efficacy of deformity correction between long- and short-segment fusion.

Variable	Short_segment (rate_IQR)	Long_segment (rate_IQR)	Short_segment_improvement_p	Long_segment_improvement_p	Between_group_p	Between_group_delta
Coronal_plane_balance_improvement_rate	46% (9.5%, 63.3%)	−5.7% (−45.9%, 34.5%)	0.386	0.8	0.525	0.175
Major_curve_Cobb_improvement_rate	85.7% (71.1%, 93.5%)	68.6% (64.4%, 76.7%)	< 0.001	0.022	0.11	0.399
Head_side_compensatory_Cobb_improvement_rate	54.5% (42.5%, 84%)	−3.4% (−16.7%, 0%)	< 0.001	0.207	< 0.001	0.91
Rear_side_compensatory_Cobb_improvement_rate	86.4% (62.2%, 93.1%)	63.3% (−9.4%, 77.5%)	< 0.001	0.108	0.127	0.391
Coronal_sacral_inclination_angle_improvement_rate	58.8% (40%, 100%)	50% (0%, 100%)	0.01	0.855	0.716	0.113
Segmental_kyphosis_improvement_rate	40.8% (13.2%, 64.2%)	54.5% (−8.3%, 61.2%)	0.011	0.271	0.692	−0.104
Sagittal_plane_balance_improvement_rate	57.1% (20.8%, 76.5%)	19.8% (−28.6%, 70%)	0.932	0.529	0.508	−0.2
Pelvic_tilt_angle_improvement_rate	20% (−10%, 55.9%)	60% (50%, 80%)	0.759	0.134	0.1	0.545
Thoracic_kyphosis_angle_improvement_rate	17.4% (−28.6%, 53%)	40.4% (25%, 43.1%)	0.144	0.295	0.851	0.058
Thoracolumbar_kyphosis_angle_improvement_rate	45.3% (21.8%, 85.3%)	53.8% (45.3%, 63.9%)	0.037	0.022	0.659	−0.118
Lumbar_lordosis_angle_improvement_rate	9.7% (−29.1%, 40.7%)	13.1% (−0.8%, 22.5%)	0.338	0.31	0.902	−0.037
Sagittal_sacral_inclination_angle_improvement_rate	−2.8% (−19%, 28.9%)	0% (−8.9%, 10.8%)	1	0.916	0.769	−0.081

**Table 4:** Differential efficacy of in maintenance of deformity correction between long- and short-segment fusion.

Variable	Short_segment (rate_IQR)	Long_segment (Rate_IQR)	Short_segment_maintenance_p	Long_segment_maintenance_p	Between_group_p	Between_group_delta
Coronal_plane_balance_maintenance_rate	17.3% (−19.6%, 66.1%)	125.7% (74.4%, 150.3%)	0.004	0.855	0.152	−0.5
Major_curve_Cobb_maintenance_rate	83.3% (71.8%, 96.5%)	91.7% (68.5%, 95.5%)	0	0.036	1	0
Head_side_compensatory_Cobb_maintenance_rate	50% (19.6%, 97.6%)	45.8% (20.8%, 70.8%)	0.003	0.1	0.608	0.174
Rear_side_compensatory_Cobb_maintenance_rate	81.2% (59.8%, 97.2%)	100% (94.4%, 105%)	0.021	0.855	0.176	−0.411
Coronal_sacral_inclination_angle_maintenance_rate	55% (−6.2%, 103.6%)	14.3% (0%, 42.3%)	0.012	0.098	0.437	0.262
Segmental_kyphosis_maintenance_rate	15.5% (−47.5%, 62.3%)	75% (41%, 116.7%)	0.003	0.447	0.193	−0.357
Sagittal_plane_balance_maintenance_rate	81% (54.7%, 107.7%)	−49.3% (−71.7%, 26.6%)	0.154	0.423	0.272	0.467
Pelvic_tilt_angle_maintenance_rate	41.7% (−93.6%, 152.5%)	0% (−73.1%, 44.8%)	0.262	0.1	0.552	0.25
Thoracic_kyphosis_angle_maintenance_rate	16% (−33.3%, 78.6%)	101.4% (59.3%, 132%)	0.007	1	0.074	−0.51
Thoracolumbar_kyphosis_angle_maintenance_rate	57.3% (16.7%, 70.3%)	54.8% (10.7%, 76.7%)	0.009	0.093	0.971	0.021
Lumbar_lordosis_angle_maintenance_rate	53.8% (18.6%, 71.2%)	55.6% (38.9%, 89.7%)	0.001	0.418	0.57	−0.179
Sagittal_sacral_inclination_angle_maintenance_rate	85.4% (55.9%, 106.2%)	−80% (−90%, 29.4%)	0.295	0.423	0.24	0.458

**Figure 2:**
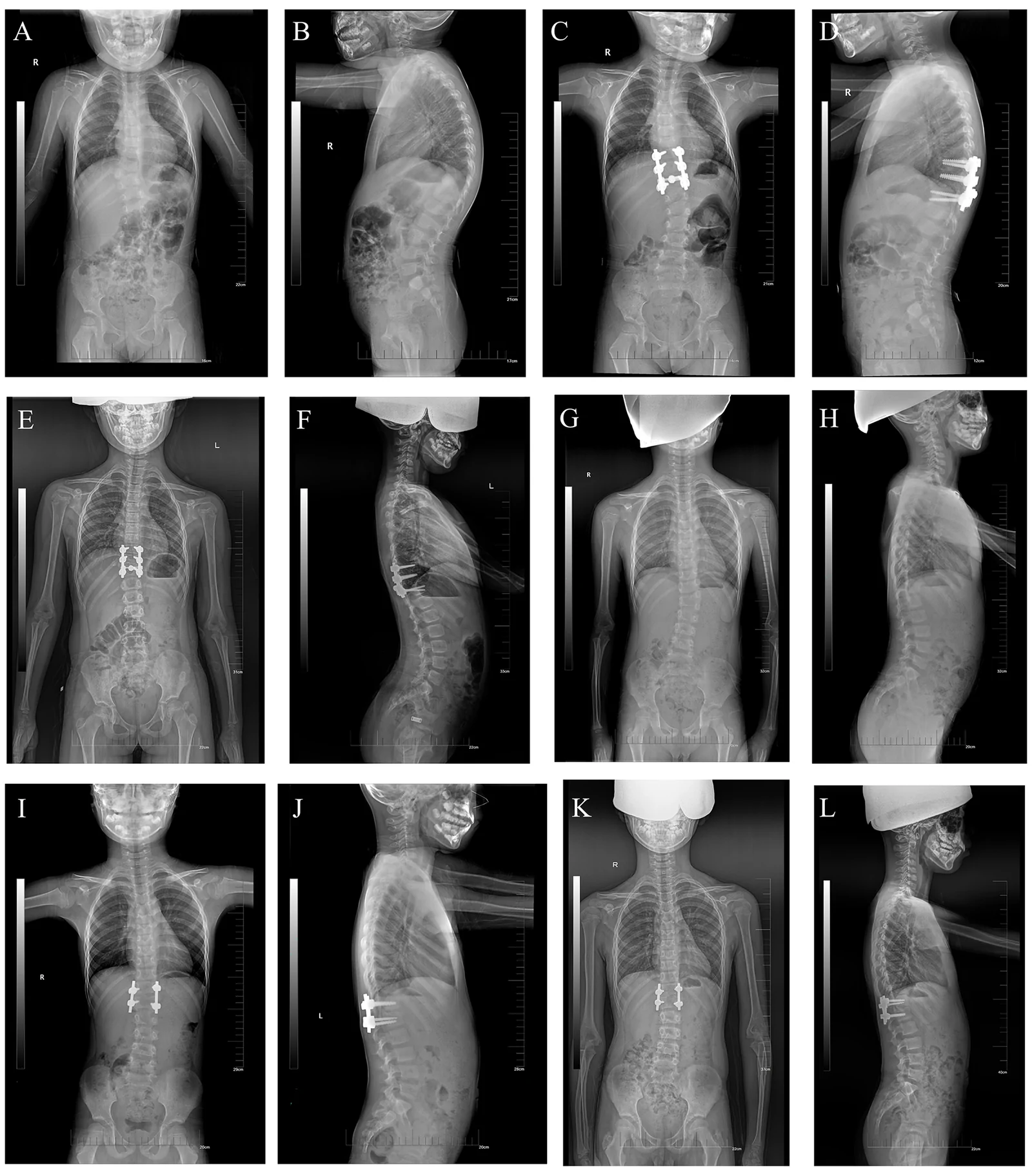
Imaging of different segmental fusion in congenital hemivertebra deformity. All patients were diagnosed with congenital hemivertebra deformity and underwent one-stage posterior hemivertebra resection and pedicle screw fixation in our center. **(A, B)** Preoperative anteroposterior and lateral radiographs of a 3.17-year-old female patient. **(G, H)** Preoperative anteroposterior and lateral radiographs of a 6.67-year-old male patient. **(C, D, I, J)** Radiographs were taken at the follow-up 1 month after the operation, and the flexion was corrected after the application of long- or short-segment fusion. **(E, F, K, L)** Follow-up images 3 years after surgery, which showed no significant progression of scoliosis after correction.

Analysis of early postoperative improvement rates showed that both groups demonstrated strong orthopedic abilities, but different areas of strength (Figure 2). The improvement of head side compensatory Cobb in SSF group was significantly better than that in LSF group (54.5 vs. −3.4%, *p* < 0.001, Cliff's delta = 0.91). The improvement of major curve Cobb was also significant in the LSF group (68.6%, *p* = 0.022). Intra-group analysis further confirmed that most coronal parameters (major curve Cobb, head side compensatory Cobb, rear side compensatory Cobb) and TLK improvement were statistically significant in SSF group (*p* < 0.05).

The maintenance rate showed that SSF group had significant maintenance effects on major curve, head side compensatory curve, rear side compensatory curve, coronal sacral inclination, segmental kyphosis, TLK and LL (*p* < 0.05). In LSF group, only major curve Cobb was significantly maintained (91.7%, *p* = 0.036). Although there was no significant difference between the two groups, the effect size showed that the long segment group had significantly higher coronal balance (125.7 vs. 17.3%; Δ = −0.50), segmental kyphosis (75.0 vs. 15.5%; Δ = −0.36), and thoracic kyphosis (101.4 vs. 16.0%; Δ = −0.51) showed a trend of better maintenance than SSF group (more than moderate effect).

### 3.3 SPs as radiographic features for predicting fusion: parameter screening, threshold selection and efficacy verification

Three SPs were selected as candidate features to distinguish long/short segment fusion strategies based on a weighted composite score of the effect size of preoperative, improvement rate, and maintenance rate (Cliff's delta; Table 5). PT (composite score = 0.456) and TLK (0.405) were high priority predictors, and LL (0.389) was medium priority predictor.

**Table 5:** Candidate features and their composite scores for single-factor screening of long- or short-segment fusion strategies.

Variable	Composite_score	Baseline_delta	Improvement_delta	Maintenance_delta	Direction_status	Priority
Pelvic_tilt_angle	0.45575	−0.47	0.545	0.25	Direction criteria met	High priority
Thoracolumbar_kyphosis_angle	0.40525	0.621	−0.118	0.021	Direction criteria met	High priority
Lumbar_lordosis_angle	0.3889	0.588	−0.037	−0.179	Direction criteria met	Medium priority
Coronal_sacral_inclination_angle	0.29075	−0.372	0.113	0.262	Direction criteria met	Low priority
Head_side_compensatory_Cobb	0.286	−0.054	0.91	0.174	Direction criteria met	Low priority
Sagittal_sacral_inclination_angle	0.24375	0.258	−0.081	0.458	Direction criteria met	Low priority

Through multi-weight sensitivity analysis, a stable weight range common to all features was determined (accuracy weight: 0.4–0.6; coverage weight: 0.3–0.5), and the threshold selection of each feature remained stable within this range (Figures 1B–E). Based on the stability interval, the optimal decision threshold and its performance index of each prediction feature were further determined (Table 5).

TLK was identified as the most predictive single indicator. Both accuracy_practical (accuracy 0.4 + coverage 0.55+ sample size 0.05) and balanced_practical (accuracy 0.5 + coverage 0.45 + sample size 0.05) strategies showed the same predictive power. When ≥36° was selected as the threshold, it was considered to be the best strategy to cover 42.9% of LSF cases while maintaining 100% accuracy (Figure 1G). However, given its limited coverage, it is not recommended to use this indicator alone for decision making. As a complement, 100% case coverage was achieved when ≥10° was chosen as the threshold, providing an alternative for screening purposes.

The optimal threshold of LL under both strategies was ≥66°, corresponding to an accuracy rate of 80% and a coverage rate of 57.1% (Figure 1F). The threshold range (≥61–66°) given by different weight strategies was relatively stable (Table 6). PT failed to screen a stable threshold with sufficient discrimination power, and it is not recommended to be used alone (Figure 1H).

**Table 6:** Feature thresholds and their predictive performance.

Features	Recommended_thresholds	Accuracy	Coverage	Strategy	Optimal_strategy	Suggestions
Thoracolumbar_kyphosis_angle	≥36	100%	42.90%	Accuracy_practical	Best_choice	Not_recommended_to_use_alone
	≥36	100%	42.90%	Balanced_practical		
	≥10	35%	100%	Coverage_practical		
Pelvic_tilt_angle	No_specific_threshold	/	/	/		
Lumbar_lordosis_angle	≥66	80%	57.10%	Accuracy_practical	Best_choice	Use with caution–should be combined with other measures
	≥66	80%	57.10%	Balanced_practical		
	≥61	62.50%	71.40%	Coverage_practical		

### 3.4 Fusion strategy prediction model based on single sagittal factor decision tree

**Figure 3:**
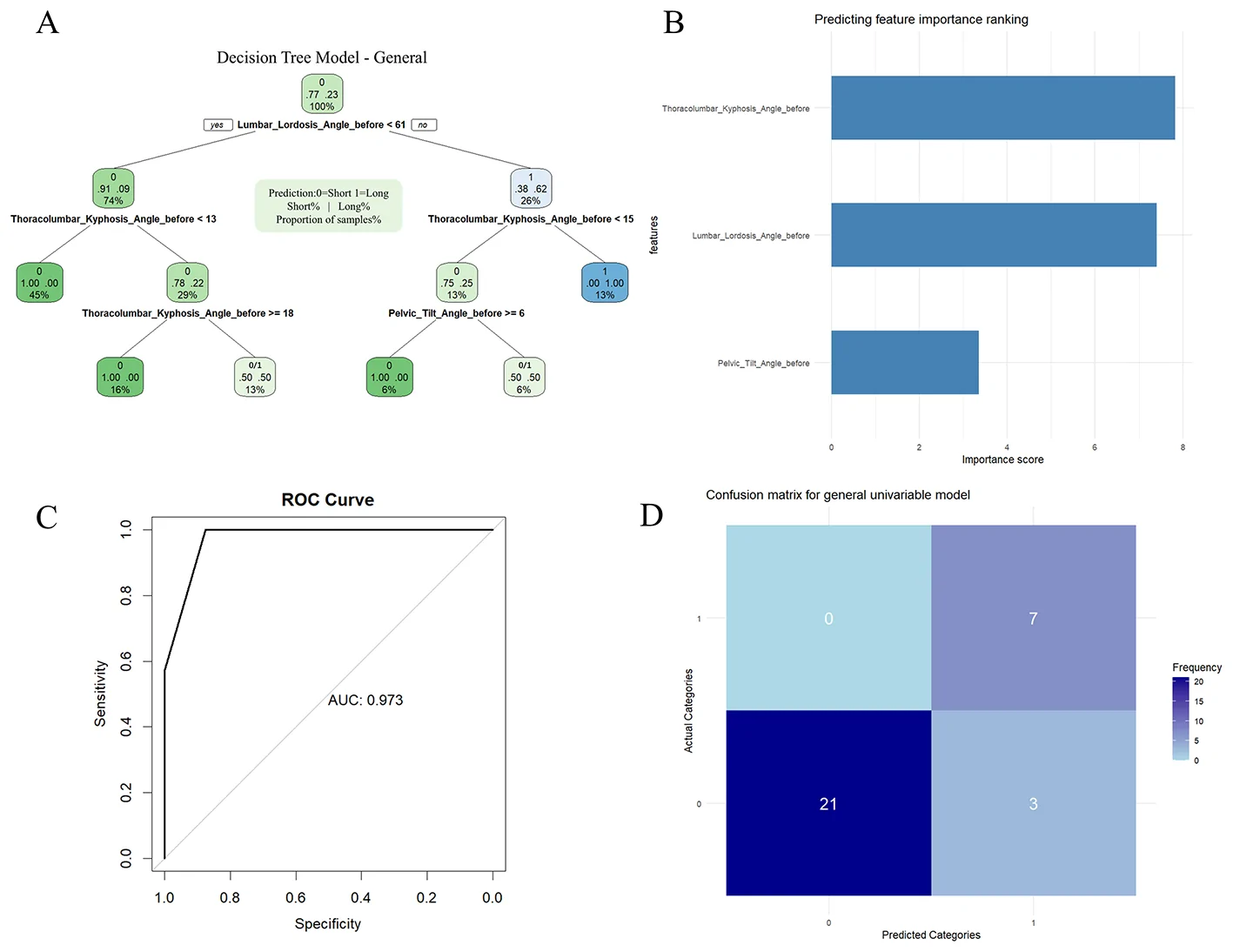
Construction and performance verification of general univariable decision tree model. **(A)** Decision-making scheme of the general univariable decision tree model. **(B)** The proportion of importance of the three key features in the general univariable model. **(C)** The ROC curve of the general univariable model. **(D)** The confusion matrix of the general univariable model.

The decision tree structure and splitting rules based on single factors are shown in Figure 3A. LL (critical value = 60.5°) was used as the primary split point. When LL was less than 60.5°, SSF was recommended when TLK was less than 12.5° or more than 17.5°, and could not be clearly recommended when TLK was between the two. For LL ≥60.5≥, if TLK ≥15°, LSF is recommended. If PT is less than 15°, it can be further subdivided according to PT: when PT is more than 6°, SSF is recommended, and when PT is less than 6°, it cannot clearly recommended.

The decision tree model showed good prediction performance (Table 7). The overall accuracy was 90.3% (95% CI: 0.774–1). The sensitivity and precision of the model for LSF cases were 100 and 70.0%, respectively. The specificity of SSF was 87.5% (95% CI: 0.720–1). The area under the ROC curve (AUC) was 0.973 (95% CI: 0.919–1; Figure 3C). The confusion matrix (Figure 3D) further visualized the accurate classification of the model. Feature importance analysis (Figure 3B) confirmed TLK as the most important predictor.

**Table 7:** Comparison of the comprehensive performance of the general univariable model with the stratified model.

Indicators	General_model (95% CI)	Classification_model (95% CI)	Improvement
Accuracy	0.903 (0.774, 1)	0.935 (0.839, 1)	0.032
Long-segment recall	1 (1, 1)	1 (1, 1)	0
Long-segment precision	0.7 (0.400, 1)	0.778 (0.500, 1)	0.078
Short-segment specificity	0.875 (0.720, 1)	0.917 (0.800, 1)	0.042
AUC	0.973 (0.919, 1)	0.982 (0.933, 1)	0.0093
Rate of change in treatment decisions	/	9.70%	0.097

### 3.5 The decision tree model with hemivertebra position stratification showed better performance

**Figure 4:**
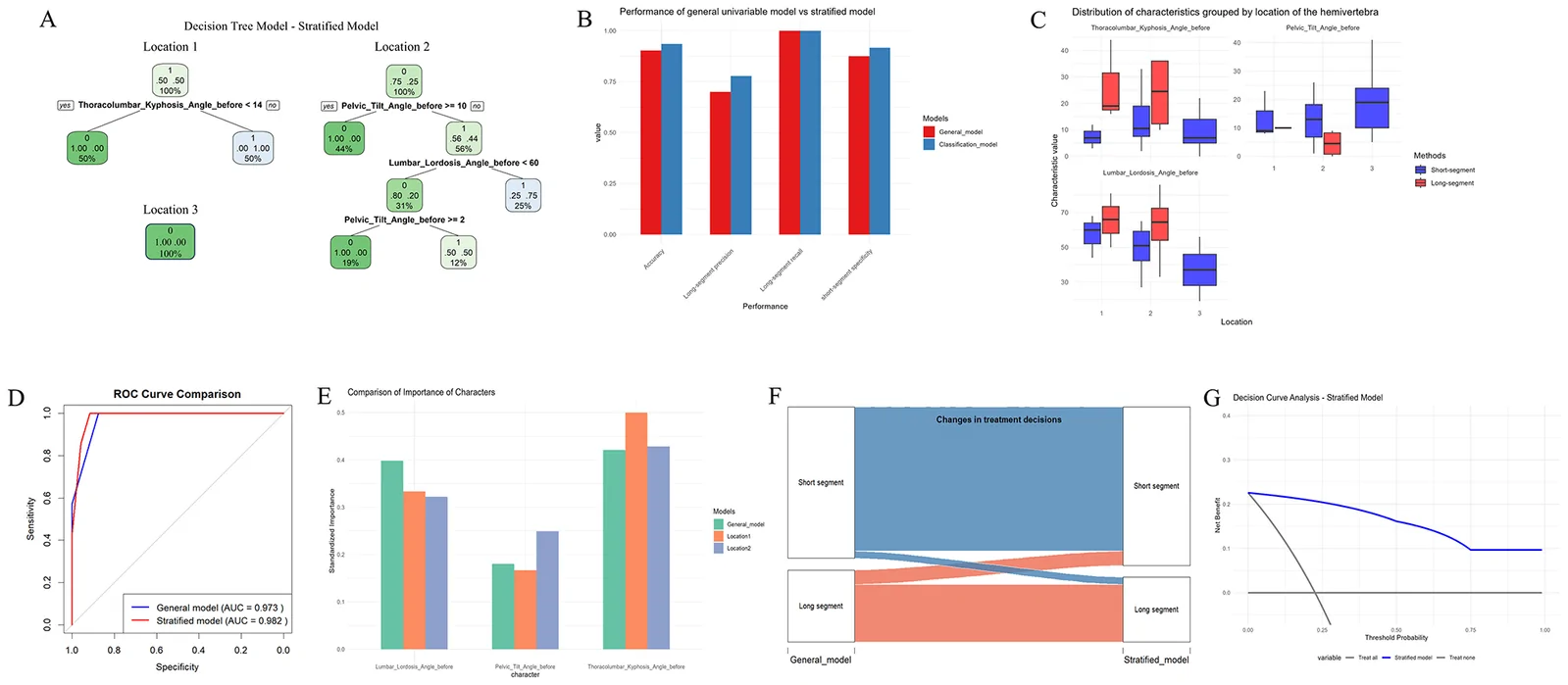
Construction of stratified decision tree model and its performance verification in the selection of hemivertebra fusion strategies. **(A)** Decision-making scheme of the stratified decision tree model. **(B)** Performance comparison between stratified model and generic univariate model in identifying different types of patients. **(C)** The ability of different core features to distinguish fusion strategies for patients with different hemivertebra locations. **(D)** ROC curves of the stratified model and the general univariable model. A higher area under the curve (AUC) value indicates a better model performance. **(E)** The proportion of importance of the three key features in different models. **(F)** Number of patients whose fusion strategy changed after using different models. **(G)** Calibration curve for the predicted probabilities of the model output.

After stratified modeling according to the position of the hemivertebra, the decision rules showed differences (Figure 4A). At the thoracic level (T1–T10), only TLK was used as the basis for decision: < 14° SSF was recommended, ≥14° LSF was recommended. The thoracolumbar/lumbar segments (T11-L5) were stratified by multiple factors. first, PT (9.5°) was used as the node, and when PT was less than 9.5°, LL (60°) was further divided. There were uncertain nodes in some branches.

The performance of the stratified model has been further enhanced (Table 6), with an accuracy rate of 93.5% (95% CI: 0.839–1), the LSF sensitivity remaining at 100% (95% CI: 1–1), the precision rising to 77.8% (95% CI: 0.500–1), and the AUC increasing to 0.982 (95% CI: 0.933–1; Figures 4B and D, Table 7). The core predictive features showed good discrimination across subgroups of hemivertebra location (thoracic, thoracolumbar, and lumbar; Figure 4C). The TLK remained the most important feature (Figure 4E). The McNemar test showed that the performance difference between the stratified model and the general model was not statistically significant (*p* = 0.903). The application of the stratified model can change the treatment decisions of 9.7% of the cases (Figure 4F). DCA was performed to evaluate the clinical net benefit of the model (Figure 4G). The results demonstrated that across a broad range of clinically reasonable threshold probabilities (approximately 0.2–0.8), the net benefit of the stratified model consistently exceeded that of both the “Treat All” and “Treat None” extreme strategies.

## 4 Discussion

In surgical treatment of congenital scoliosis hemivertebra deformity, LSF or SSF strategy always faces a choice, which can achieve the ideal overall spinal balance while minimizing surgical trauma and preserving motor function (10, 11). Although the evaluation of radiography is closely related to surgical selection and clinical outcomes, there are still challenges in quantifying and translating it into a decision tool that can directly guide selection. Based on the retrospective data of 36 patients from a single center, this study used machine learning algorithms to mine the core radiographic parameters and construct a clinical decision model.

Due to the current lack of a gold standard, in order to determine whether there was a systematic bias in clinicians' selection of fusion segments, we first compared the radiographic characteristics of patients in LSF or SSF group before operation and at the last follow-up. The core SPs such as TLK and LL in LSF group were significantly higher than those in the short fusion group (*p* < 0.05). This suggests that patients with more severe disease and more pronounced sagittal imbalance chose longer segments of fusion, which is consistent with the basic logic of clinical decision making and is consistent with the retrospective results of other centers (12, 13). At 1 month after operation and last follow-up, the differences of above parameters between the two groups disappeared (*p* > 0.05), and the effect size decreased to a negligible level (Cliff's delta < 0.147). LSF corrected the initial more severe deformity to a level comparable to that of SSF group by more extensive fusion and did not lead to overtreatment.

Further analysis of the efficacy showed that the average correction rate of Cobb of the major curve was more than 80% in both groups, and the maintenance rate was more than 90%. There was no significant correction loss at the last follow-up. No internal fusion failure, crankshaft phenomenon or secondary operation occurred. There was no correction failure due to insufficient fusion in SSF group, and there was no increase in the risk of complications due to unnecessary lengthening of fusion in the LSF group, which ruled out the possibility of under-treatment. Therefore, the segment selection based on preoperative radiographic parameters was proved to be reasonable and effective at the outcome level, which provided a solid clinical basis for supervised learning modeling in this study.

This study found that LL, TLK and PT were the most discriminative core parameters to distinguish the two types of fusion strategies. Interestingly, the three metrics that ultimately dominated the model decision were all SPs. This result may be highly compatible with the biomechanical theory of spinal-pelvic balance. In recent years, more and more attention has been paid to the recovery of sagittal balance in the correction of scoliosis (14–16). It is currently believed that the onset and progression of scoliosis is due to asymmetric regulation of vertebral growth caused by changes in the load distribution of the spine (17). Sagittal instability directly disrupts the conduction and energy efficiency of the gravity line, which has an intrinsic drive for continuous progression (18). The thoracolumbar junction (T11–L2) is the mechanical hub for the transition of the spine from the relatively rigid thoracic spine to the active lumbar spine, and the physiological kyphosis is minimal. Once kyphosis occurs in this region, the local and even global biomechanical moment arm will be significantly enlarged, and the sagittal balance will be further damaged (19, 20). In this study, TLK ranked first in feature importance, and similarly, a study based on logistic regression also included TLK as one of the indicators (13). PT reflects the connection between the trunk gravity line and the lower limbs in the upright position, and is a key indicator of the stability of the overall balance chain (21, 22). Previous studies have confirmed that preoperative PT is a significant independent risk factor for scoliosis progression (23). Therefore, TLK, LL and PT are not only the measurement of local deformity, but also highly sensitive indicators of global sagittal instability. After fusion, the balance of all segments of the spine is redistributed (19). Therefore, the model takes these three indicators as decision nodes in order to accurately capture the core pathophysiology of hemivertebra deformity.

Despite the single-center nature of this study, the decision rules generated by our model are consistent with previously published evidence from independent cohorts. Peng et al. (13) in a retrospective analysis of patients undergoing hemivertebra resection, identified preoperative thoracic kyphosis ≤ 30.2° as a predictor for suitability of SSF, demonstrating that sagittal alignment is a key determinant of fusion length selection across different institutions. Similarly, Gomez et al., (24) in a multi-center study, reported that patients undergoing LSF had significantly greater preoperative segmental kyphosis (*p* = 0.046). Notably, LSF group in that study also presented with larger preoperative T2–T12 kyphosis (*p* = 0.013), further corroborating the relationship between sagittal deformity severity and the selection of LSF. Moreover, the incorporation of LL and PT as secondary decision nodes aligns with the Roussouly classification framework, which emphasizes that spinal-pelvic parameters are fundamental to assessing global sagittal balance and guiding surgical planning. A meta-analysis further confirmed that restoring patients to their ideal Roussouly type significantly reduces mechanical complications (OR = 0.22, *p* < 0.001), underscoring the clinical relevance of incorporating LL and PT into fusion strategy decisions (25). Thus, while our model represents a quantification of our institutional approach, its core decision logic is grounded in well-established biomechanical principles and externally validated evidence.

The clinical value of the prediction model needs to be verified by postoperative correction effect and long-term stability maintenance. In terms of short-term improvement, the model-recommended strategy was able to produce the expected immediate corrective effect. The SSF cases showed excellent improvement in the major curve Cobb (85.7%) and the head side compensation curve (54.5%), while LSF cases showed significant improvement in TLK (53.8%). The long-term maintenance rate of thoracic kyphosis (101.4%) and segmental kyphosis (75.0%) in LSF group was better than that in SSF group, showing its advantage in the long-term stability of complex sagittal deformity. The coronal parameters such as the maintenance rate of the major curve Cobb (83.3%) also remained stable in SSF group, which confirmed its reliability in the corresponding deformity types. This consistency from short-term to long-term fully validates the effectiveness and reliability of the strategy recommended by the model in clinical practice.

The model showed significant advantages in decision robustness. Weight sensitivity analysis showed that the core structure of the model and the key threshold remained stable within a reasonable weight range. Subgroup analysis showed that all the three core predictors showed significant discrimination between the long and short segments in patients with different hemivertebral position, although the differences in effect sizes. This suggests that the three core parameters may go beyond regional anatomical differences and collectively point to the assessment of the overall “spinal-pelvic” balance.

In terms of clinical value, the 100% LSF sensitivity of the model ensures that all complex and severe deformity cases that truly require LSF can be effectively identified and undertreatment can be avoided to the greatest extent. At the same time, the high specificity reduces unnecessary long segment fusion, which correspondingly shorts the operation time, reduces bleeding, reduces the cost of implants and the risk of complications (24, 26). The “uncertainty node” in the path is the mechanism for identifying complex cases and prompting prudent evaluation. Although the McNemar test did not show a significant classification difference between the stratified model and the general model (*p* = 0.903), the stratified model increased the LSF accuracy from 70.0 to 77.8%, significantly reducing the misjudgment rate without sacrificing the recognition rate, which has clear clinical significance. DCA results indicate that, within this threshold range, using the model to guide decisions can effectively reduce unnecessary procedures or avoid missing high-risk patients without increasing additional clinical risks, confirming its reliable potential for clinical translation.

We acknowledge that the present model was developed and internally validated using data from a single institution, which inevitably introduces center-specific practice patterns. Thus, multi-center external validation is critically needed to confirm the generalizability of our decision thresholds before they can be widely adopted.

Several other limitations should also be considered. First, the sample size was relatively limited (*n* = 36), particularly for the LSF subgroup (*n* = 7). Although LOOCV and weight sensitivity analysis supported the structural stability of the model, the wide CIs for sensitivity (e.g., 59.0%−100%) honestly reflect the uncertainty inherent in the data and underscore the need for larger confirmatory cohorts. Second, the retrospective design cannot fully eliminate selection bias, and the “uncertainty nodes” in the decision tree require further validation with more data. Third, potential confounders such as bone maturity, bone quality, neurologic status, and growth potential were not included; although previous studies have attempted to use Risser sign combined with SPs to predict scoliosis progression (27), how to accurately quantify spinal growth potential and its dynamic process is still a key direction for future research. Finally, excluding patients with severe postoperative complications introduced idealized cohort bias, meaning our model was trained on uneventful cases and is thus most directly applicable to low-risk patients (e.g., without neurological deficits). For high-risk patients, the model's recommendations should serve as a reference, not a directive, and must be overlaid with clinical judgment regarding additional risk factors.

## 5 Conclusion

Based on clinical radiography data, this study constructed a decision tree model focusing on three core SPs, which provided an objective and accurate decision support tool for the surgical treatment of hemivertebra deformity. The model integrated the preoperative baseline, postoperative correction and long-term maintenance trajectory, forming a complete evidence chain from patient selection, preoperative planning to prognosis evaluation. The uncertainty nodes in the model are complex situations that need careful evaluation. Clinicians can start additional evaluation processes, such as three-dimensional image reconstruction, radiography in other positions, or enhanced follow-up, so that the decision tree can be embedded and optimize the existing diagnosis and treatment pathways. Through multi-center prospective validation, larger sample size, and fusion of multimodal data such as MRI and growth potential assessment, it is expected to further iteratively optimize the model, and finally realize the precise surgical treatment of congenital scoliosis.

## Data Availability

The datasets presented in this article are not readily available due to ethical restrictions. The datasets analyzed during this study are available from the corresponding author upon reasonable request, subject to Ethics Committee approval. Requests to access the datasets should be directed to Fuyong Zhang, fuyongzhang2023@163.com.

## Supplementary material

Supplementary Table S1 The preoperative, postoperative (1 month) and the last follow-up radiographic data of the two groups.
